# Two visual working memory representations simultaneously control attention

**DOI:** 10.1038/s41598-017-05865-1

**Published:** 2017-07-21

**Authors:** Yanan Chen, Feng Du

**Affiliations:** 1 CAS Key Laboratory of Behavioral Science, Institute of Psychology, Chinese Academy of Sciences, Beijing, 100101 China; 2 Psychology Department, University of Chinese Academy of Sciences, Beijing, 100101 China; 3 Institute of Behavior and Psychology, Henan University, Kaifeng, 475004 China

## Abstract

It has been proposed that only one visual working memory (VWM) representation can be activated to influence perception directly, whereas other VWM representations are accessory items which have little influence on visual selection. The sole active VWM representation might reflect a fundamental bottleneck in the information processing of human beings. However, the present study showed that each of two VWM representations can capture attention and interfere with concurrent visual search. In addition, each of two VWM representations can interfere with concurrent visual search as much as can a single cued VWM representation. Moreover, when two memory-matching distractors appear in visual search, two VWM representations produce a larger memory-driven capture effect than a single memory-matching distractor. Thus, two VWM representations can simultaneously control attention.

## Introduction

The contents of visual working memory (VWM) play a critical role in deploying attention and bias perceptual processing toward memory-matching items^1^. In a visually crowded world, for example, a VWM representation of a target, usually referred to as the target template or attention template, can efficiently guide visual search in a top-down fashion^2^. Studies have shown that neural responses in the visual cortex are biased toward an object matching the target template^3, 4^. In addition, VWM contents that are irrelevant to the target template can also involuntarily capture attention. For instance, if participants are required to remember an item while searching for another target, the concurrent visual search is delayed when a memory-matching distractor is present in the search display, relative to when a new salient distractor is present in the search display^5–9^.

Humans can, on average, hold about 3–4 items in visual working memory^10^. However, it has been proposed that VWM representations have a different status^11^ and that only one item in working memory can be attended to at a time^12^. Oliver and colleagues^13^ proposed two types of VWM representation. One type is an active memory item and it has direct access to perception, thus serving as an attention template. The other type of VWM representation involves accessory memory items which are passively stored in VWM, but they have little influence on visual selection. Importantly, only one item in VWM can be activated at a time to serve as an attention template^13, 14^. Consistent with this proposition, a target template consumes the sole active slot in VWM when targets vary from trial to trial, thus an irrelevant stimulus that matches other accessory contents in VWM cannot interfere with concurrent visual search^15, 16^. However, when the target is constant from trial to trial, the target template is stored in long-term memory instead of VWM after a few trials^17, 18^; consequently, another sole item in VWM becomes automatically activated to bias perception^15, 18^. For example, when the target is constant across trials, a distractor that matches the presumably sole active item in VWM delays concurrent visual search relative to a memory-unmatched distractor^7–9^. However, even with a constant target, two VWM items would compete with each other and eventually both would become accessory VWM representations. For example, if participants are required to memorize two colors in VWM, a distractor matching either of those colors has no influence on concurrent visual search^14^.

The sole active VWM representation might reflect a fundamental bottleneck in human information processing. However, this idea is not supported by some recent findings that irrelevant stimuli matching either of two target colors can involuntarily capture attention^19–23^. For example, when participants searched for either of two specifically colored targets among other colored distractors, non-predictive cues matching either target color produced a significant spatial cueing effect, while irrelevantly colored cues did not^24^. These studies consistently show that attention can be under the simultaneous control of two target templates for color. Although targets changed from trial to trial^20, 24^ or even varied within a single trial^23^, there were only two possible target colors, thus resulting in two constant target templates in those studies. Since previous studies have shown that the a constant target is initially stored in working memory but rapidly transfer to long-term memory after a few trials^17, 18^, two constant target templates are also likely to be stored in long-term memory rather than VWM. Thus the previous finding of the simultaneous control of attention by two target colors may not due to two active presentations in VWM. The present study aimed to examine whether two VWM representations can be simultaneously activated to influence perception.

If only one VWM representation can be activated, then there should be no memory-driven attentional capture when VWM is loaded with two colors^14^. Admittedly, even with a single active WM slot, two VWM representations might also be alternately activated to guide attention on a trial. As a result, the combined memory-driven capture effect for two VWM representations should be equal to that of a single cued memory item. Furthermore, if two VWM representations are alternately activated with unequal probability, at least one of them should produce a memory-driven capture smaller than that for a single cued memory item. In contrast, if there are multiple active slots in VWM, then two VWM representations can be simultaneously activated to guide attention, irrelevant distractors matching either of the two VWM representations should capture attention. As a result, the combined memory-driven capture effect for two VWM representations should be significantly larger than that of a single cued memory item. In addition, each of two VWM representations is supposed to capture attention as much as a single cued object. Since conjunctions of multiple features have enhanced neural correlates of VWM representations compared with a single color feature^25^, multiple VWM representations of conjunctions are likely to capture attention. The present study aimed to examine whether two VWM representations can simultaneously guide attention and interfere with concurrent visual search when participants are required to memorize two items with feature conjunctions.

## Results

Only when responses in the search and the memory task are both correct, reaction times for search task were analyzed. RTs data was trimmed based on a cutoff value of ±3 SD from the mean per participant. All results are based on the trimmed data.

### Experiment 1

**Figure 1:**
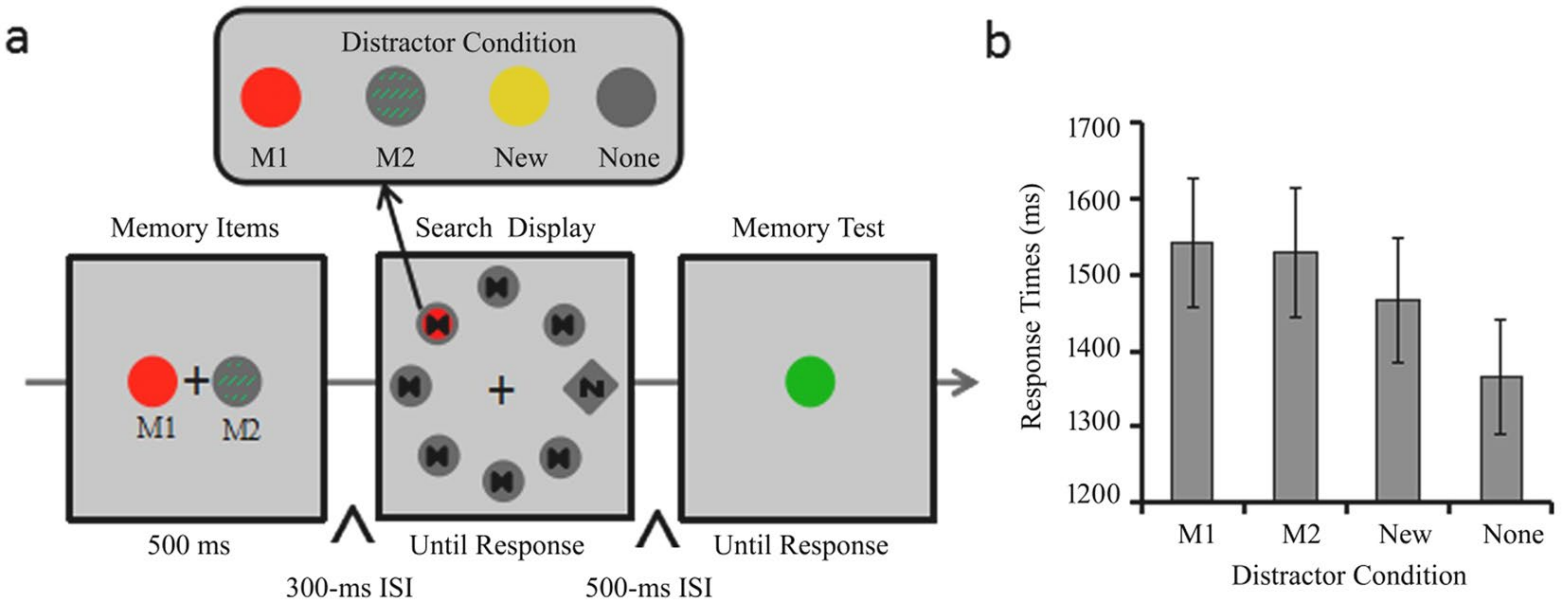
Trial sequences and results of Experiment 1. (**a**) The sequence of events with four possible distractor conditions in a trial of Experiment 1 (the M1 distractor condition: the disk’s color and texture was the same as the left memorized item; the M2 distractor condition: the disk’s color and texture was the same as the right memorized item; the New distractor condition: the disk was a new solidly colored disk; the None distractor condition: all seven disks were gray). (**b**) RTs for the search task as a function of distractor condition in Experiment 1. Error bars indicate ±1 SE.

The ***Experiment 1*** aimed to examine whether two VWM representations can be automatically activated to interfere with a concurrent visual search. Participants were required to memorize two objects by their specific conjunction of color and texture before starting a visual search task which was completely irrelevant to the memory task (See method and Fig. 1a for details). Data trimming resulted in a loss of 1.87% of trials. Figure 1b shows the mean RTs as a function of distractor condition in Experiment 1. There was a main effect of distractor condition, *F* (3, 69) = 21.693, *p* < 0.001, *η*_*p*_^2^ = 0.485. Further pairwise comparisons with Bonferroni adjustment revealed that the RTs of the M1 condition and the M2 condition were significantly longer than the RTs of the New distractor condition (*ps* < 0.05). Also, the RTs of the M1 condition and the M2 condition were significantly longer than the RTs of the No distractor condition (*ps* < 0.001). And the New distractor condition produced significantly longer RTs than the No distractor condition (*p* < 0.01). The same analysis on search accuracy showed no such effects, *F* (3, 69) = 1.266, *p* = 0.293, *η*_*p*_^2^ = 0.052 (see Table 1 for search accuracy).

**Table 1:** Average RTs and mean accuracy for the Search Task, and the mean accuracy for the Memory Task in Experiments 1, 2, 3, 4 and 5.

Experiments	Distractor Condition	Search RT (ms)	Search Accuracy (%)	Memory Accuracy (%)
Experiment 1	M1	1542 (419)	99.1 (2.1)	93.3 (7.4)
	M2	1528 (417)	98.7 (1.8)	92.2 (7.6)
	New	1466 (398)	98.3 (2.6)	91.4 (8.9)
	None	1365 (373)	98.4 (2.8)	92.1 (7.7)
Experiment 2	Cued	1358 (353)	97.7 (3.4)	94.8 (6.8)
Single cued memory item	Uncued	1296 (369)	97.7 (4.3)	93.8 (7.7)
(One item is 100% cued)	New	1305 (375)	97.8 (3.9)	93.3 (8.0)
	None	1198 (331)	97.7 (3.2)	94.8 (5.4)
Experiment 2	M1	2031 (756)	97.9 (3.3)	80.0 (12.1)
Two memory items	M2	2020 (801)	97.6 (3.7)	78.8 (13.3)
(Two items are not cued)	New	1932 (713)	97.5 (3.2)	77.3 (14.3)
	None	1847 (678)	97.5 (4.3)	80.0 (12.8)
Experiment 3	M1	1577 (593)	98.9 (1.9)	88.4 (9.0)
Long SOA	M2	1567 (570)	99.1 (1.7)	89.9 (7.5)
	New	1465 (467)	99.0 (1.4)	87.8 (9.7)
	None	1348 (430)	99.2 (1.2)	88.7 (7.0)
Experiment 4	Cued	995 (156)	96.6 (3.5)	94.0 (5.2)
Single cued memory item	Uncued	937 (153)	97.5 (3.1)	93.0 (5.4)
(One item is 100% cued)	New	932 (136)	97.1 (2.9)	94.2 (6.0)
	None	875 (125)	96.9 (4.0)	93.2 (6.1)
Experiment 4	M1	986 (166)	96.6 (3.3)	81.3 (13.1)
Two memory items	M2	983 (156)	95.9 (4.0)	81.3 (12.7)
(Two items are not cued)	New	936 (136)	95.8 (3.0)	81.8 (13.5)
	None	905 (133)	97.1 (3.3)	81.8 (9.9)
Experiment 5	Cued	1186 (146)	98.7 (1.6)	96.6 (4.6)
Single cued memory item	Uncued	1093 (166)	98.3 (2.2)	95.1 (4.4)
(One item is 100% cued)	New	1113 (170)	99.1 (1.3)	95.0 (4.5)
	None	1021 (161)	98.4 (2.4)	95.8 (5.4)
Experiment 5	Match-2	1301 (215)	97.8 (2.7)	82.2 (12.7)
Two memory items	Match-1	1235 (199)	97.1 (2.5)	81.9 (12.1)
	Match-0 (New)	1174 (179)	97.7 (2.1)	78.8 (11.6)
	None	1083 (155)	98.4 (1.8)	83.2 (13.5)

Data between parentheses represents SDs.

Accuracy for the memory task in Experiment 1 is also listed in Table 1. There was no significant main effect of distractor type, *F* (3, 69) = 0.793, *p* = 0.502, *η*_*p*_^2^ = 0.033. Thus, the performance of the memory test was not affected by the different distractor conditions.

### Experiment 2

Though Experiment 1 showed both M1 and M2 capture attention, they might alternately engage a single active working memory slot. If M1 and M2 share a single active WM slot, only one VWM representation (either M1 or M2) can be activated to guide attention on any given trials. As a result, the combined memory-driven capture effect for M1 and M2 (M1 + M2) should be equal to that of Cued object. Furthermore, if M1 and M2 are alternately activated with unequal probability, at least one of them should produce a memory-driven capture smaller than that for the Cued distractor. In contrast, if there are multiple active slots in VWM, then M1 and M2 can be simultaneously activated in VWM as a single Cued object does. As a result, both M1 and M2 are supposed to capture attention as much as the Cued object.

**Figure 2:**
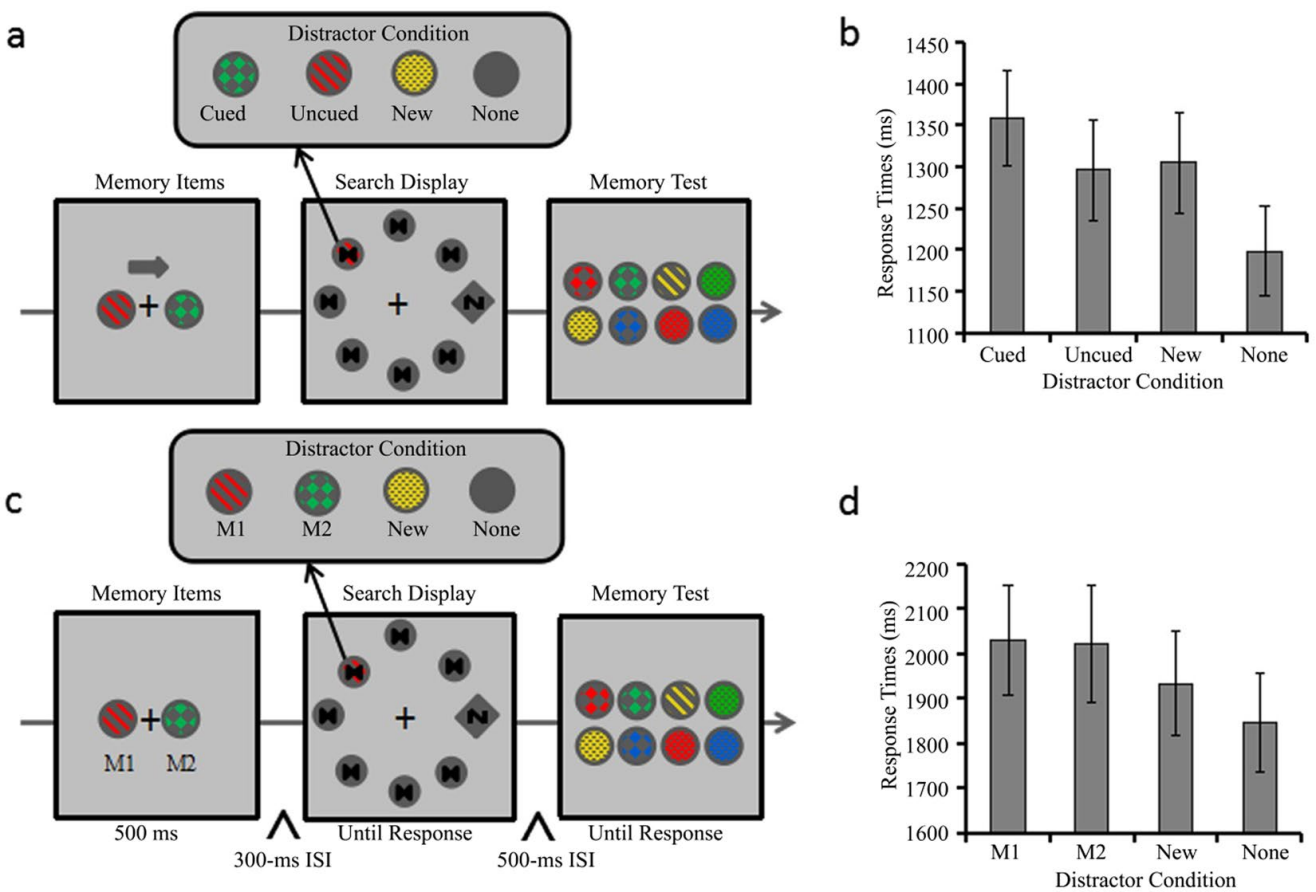
Trial sequences and results for Experiment 2. (**a**) The sequence of events with four possible distractor conditions in a trial for the single cued memory item condition (the Cued distractor condition: the disk’s color and texture was the same as the Cued memorized item; the Uncueddistractor condition: the disk’s color and texture was the same as the Uncued memorized items; the New distractor condition: the disk was in a new color and new texture; the None distractor condition: all seven disks were in gray). (**b**) RTs for the search task as a function of distractor condition in the single cued memory item condition. (**c**) The sequence of events with four possible distractor conditions in a trial of the two memory items condition (the M1, M2 and None distractor conditions were identical to those in Experiment 1, the New distractor condition was the same as its counterpart in the single cued memory item condition). (**d**) RTs for the search task as a function of distractor condition in the two memory items condition. Error bars indicate ±1 SE.

The ***Experiment 2*** aimed to examine whether M1 or M2 produce a memory-driven capture smaller than that of a single Cued object. Thus the ***Experiment 2*** asked participants to either memorize one item or two items in two separate blocks to provide a within-subject comparison. When they had to memorize one item, one of two memorized items was cued to establish a baseline level for the memory-driven captures effect by a single item in VWM. Data trimming resulted in a loss of 1.04% of trials. Figure 2b shows the mean RTs as a function of distractor condition in the single cued memory item condition in Experiment 2. There was a main effect of distractor condition, *F* (3, 111) = 24.123, *p* < 0.001, *η*_*p*_^2^ = 0.395. Further pairwise comparisons with Bonferroni adjustment revealed that the RTs of the Cued condition were significantly longer than the RTs of the Uncued distractor, the New distractor condition and the No distractor condition (*ps* < 0.05). Also, the RTs of the Uncued condition were significantly longer than the RTs of the No distractor condition (*p* < 0.001). And the New distractor condition produced significantly longer RTs than the No distractor condition (*p* < 0.001). The same analysis on search accuracy showed no such effects, *F* (3, 111) = 0.029, *p* = 0.993, *η*_*p*_^2^ = 0.001 (see Table 1 for search accuracy).

Accuracy for the memory task in the single cued memory item condition in Experiment 2 is also listed in Table 1. There was no significant main effect of distractor type, *F* (3, 111) = 2.012, *p* = 0.116, *η*_*p*_^2^ = 0.052. Thus, the performance of the memory test was not affected by the different distractor conditions.

Figure 2d shows the mean RTs as a function of distractor condition in the two memory items condition of Experiment 2. There was a main effect of distractor condition, *F* (3, 111) = 15.114, *p* < 0.001, *η*_*p*_^2^ = 0.29. Further pairwise comparisons with Bonferroni adjustment revealed that the RTs of the M1 condition and the M2 condition were significantly longer than the RTs of the New distractor condition (*ps* < 0.05). The RTs of the M1 condition and the M2 condition were significantly longer than the RTs of the No distractor condition (*ps* < 0.001). And the New distractor condition produced significantly longer RTs than the No distractor condition (*p* < 0.05). The same analysis on search accuracy showed no such effects, *F* (3, 111) = 0.370, *p* = 0.775, *η*_*p*_^2^ = 0.010 (see Table 1 for search accuracy).

Accuracy for the memory task in the two memory items condition in Experiment 2 is also listed in Table 1. There was no significant main effect of distractor type, *F* (3, 111) = 1.619, *p* = 0.189, *η*_*p*_^2^ = 0.042, indicating that the performance of the memory test was not affected by the different distractor conditions.

### Within-experiment Comparison

We developed a memory-driven capture index (MCI) to measure the interference caused by distractors. For example, the MCI of the Cued distractor in Experiment 2: MCI = (Rtcue-RTnew)/0.5*(Rtcue + RTnew). The MCI for different distractors in Experiment 2 are listed in Table 2.

**Table 2:** Average memory-driven capture index (MCI) for the different distractor conditions in Experiments 1–5.

Experiments	Distractor Condition	Memory-driven capture index	Confidence Interval of MCI
Experiment 1	M1	5.00 (6.21)	[2.38, 7.62]
	M2	4.19 (5.95)	[1.68, 6.71]
Exp2 - Single cued memory item	Cued	4.67 (7.89)	[2.07, 7.26]
	Uncued	−0.71 (6.31)	[−2.78, 1.36]
Exp2 - Two memory items	M1	4.79 (9.81)	[1.56, 8.01]
	M2	3.46 (8.23)	[0.76, 6.17]
Experiment 3	M1	5.82 (8.88)	[2.07, 9.57]
	M2	5.45 (6.95)	[2.52, 8.39]
Exp 4 - Single cued memory item	Cued	6.39 (6.88)	[4.07, 8.72]
	Uncued	0.37 (5.00)	[−1.32, 2.06]
Exp 4 - Two memory items	M1	4.95 (5.02)	[3.25, 6.65]
	M2	4.68 (6.27)	[2.56, 6.80]
Exp 5 - Single cued memory item	Cued	6.75 (6.82)	[4.21, 9.30]
	Uncued	−1.79 (5.53)	[−3.86, 0.27]
Exp 5 - Two memory items	Match-2	10.03 (8.88)	[6.72, 13.35]
	Match-1	4.95 (5.91)	[2.74, 7.15]

Data between parentheses represents SDs. MCI = (RTcue-RTnew)/0.5*(RTcue + RTnew).

If the M1 and M2 in Experiment 2 were alternating as the sole active representation in VWM, then the combined MCI for M1 and M2 (M1 + M2) should be equal to MCI for the Cued distractor in Experiment 2. For example, hypothetically, the probability of M1 to interfere with visual search is P (0 < P < 1) and its corresponding MCI should be P*MCI_(cued distractor)_, then the probability of M2 to interfere is 1-P at most and its corresponding MCI should be (1-P)*MCI_(cued distractor)_. Therefore, if M1 and M2 are alternating as a sole active representation in VWM, either M1 or M2 should produce a smaller MCI than the Cued distractor (either MCI_(M1)_ or MCI_(M2)_ < 0.5 * MCI_(cued distractor)_). However, the M1 and M2 distractors in the two memory items condition produced an MCI comparable with the Cued distractor in the single cued memory item condition, both *ts* < *0*.*55*, both *ps* *>* *0*.*58*. These results are inconsistent with the hypothesis that M1 and M2 are alternately activated in VWM.

### Experiment 3

**Figure 3:**
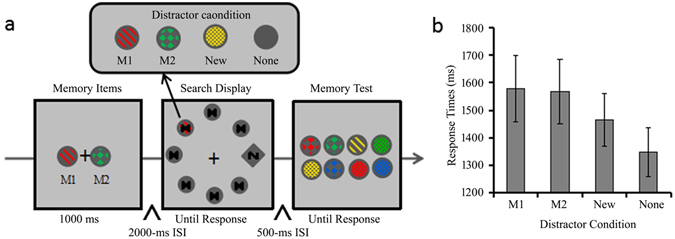
Trial sequences and results for Experiment 3. (**a**) The sequence of events with four possible distractor conditions in a trial (M1, M2, New and None distractor condition were the same as the two memory items condition in Exp 2). (**b**) RTs for the search task as a function of distractor condition. Error bars indicate ±1 SE.

Previous studies have shown that the memory-driven attentional effects decrease as the stimulus onset asynchrony (SOA) between the memory item and the search display increases^26^. Thus, with a short SOA of 800 ms in Experiments 1 & 2, the memory-driven capture effects might be slightly exaggerated. The Experiment 3 increased the SOA to 3000 ms to examine whether the two memorized feature conjunctions still capture attention with the extended SOA. Data trimming resulted in a loss of 1.69% of trials. Figure 3b shows the mean RTs as a function of distractor condition in Experiment 3. There was a main effect of distractor condition, *F* (3, 69) = 18.614, *p* < 0.001, *η*_*p*_^2^ = 0.447. Further pairwise comparisons with Bonferroni adjustment revealed that the RTs of the M1 condition and the M2 condition were significantly longer than the RTs of the New distractor condition (*ps* < 0.05). The RTs of the M1 condition and the M2 condition were also significantly longer than the RTs of the No distractor condition (*ps* < 0.001). And the New distractor condition produced significantly longer RTs than the No distractor condition (*p* = 0.001). The same analysis on search accuracy showed no such effects, *F* (3, 69) = 0.230, *p* = 0.875, *η*_*p*_^2^ = 0.010 (see Table 1 for search accuracy).

Accuracy for the memory task in Experiment 3 is also listed in Table 1. There was no significant main effect of distractor type, *F* (3, 69) = 0.689, *p* = 0.562, *η*_*p*_^2^ = 0.029. Thus, the performance of the memory test was not affected by the different distractor conditions.

### Experiment 4

**Figure 4:**
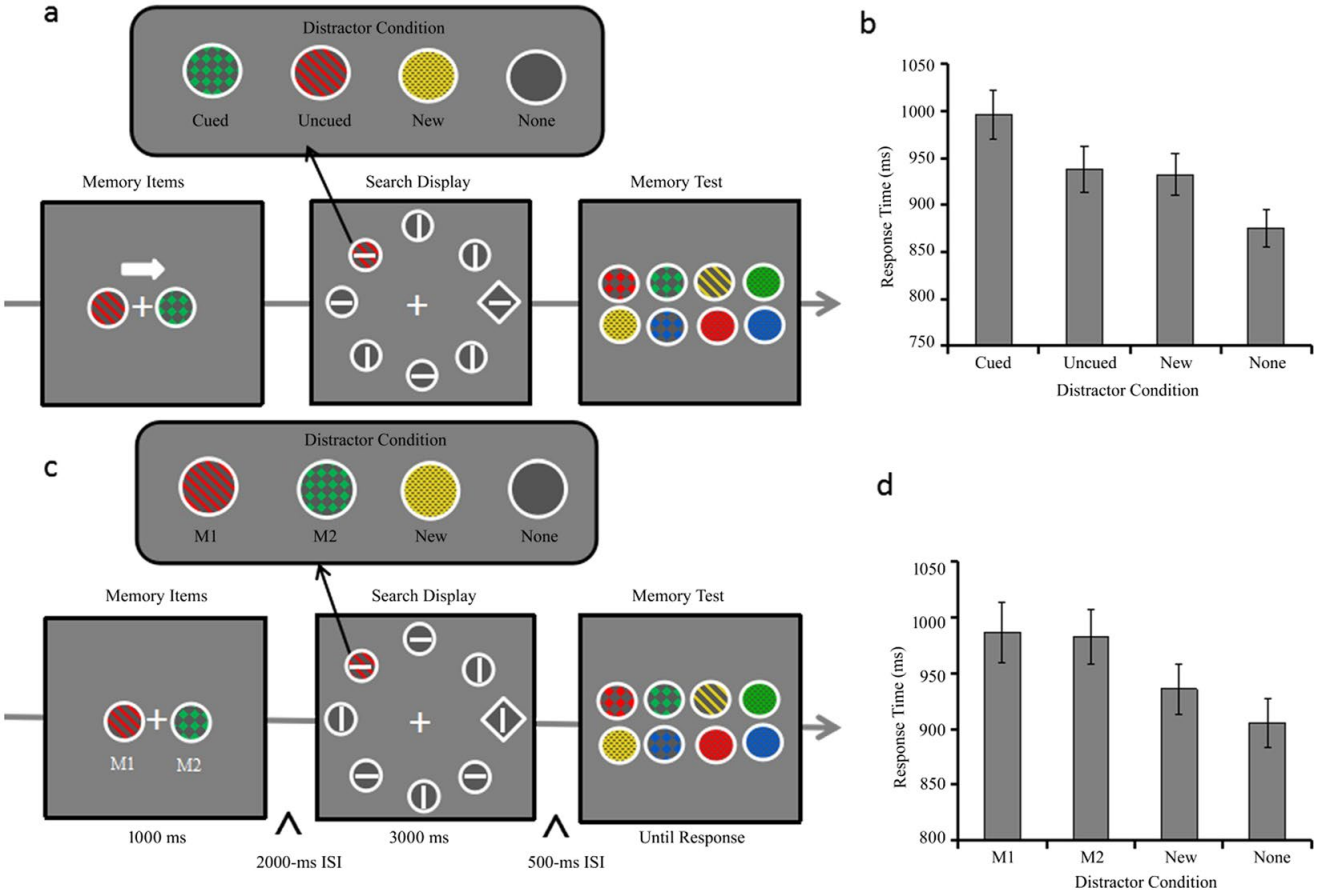
Trial sequences and results for Experiment 4. (**a**) The sequence of events with four possible distractor conditions in a trial for the single cued memory item condition (The Cued, Uncued, New and None distractor condition were the same as those in Exp 2). (**b**) RTs for the search task as a function of distractor condition in the single cued memory item condition. (**c**) The sequence of events with four possible distractor conditions in a trial of the two memory items condition (The M1, M2, New and None distractor condition were the same as those in Exp 2). (**d**) RTs for the search task as a function of distractor condition in the two memory items condition. Error bars indicate ±1 SE.

Though two visual working memory representations simultaneously capture attention in Experiments 1–3, the RTs are longer than those reported in previous studies^14^. Furthermore, the average RTs for two memory items condition are significantly longer than those for the single cued item condition in Experiment 2, which might make comparison between two conditions difficult. Thus the ***Experiment 4*** aimed to examine whether two working memory representations still capture attention when RTs are comparable to previous studies and also comparable between conditions. RTs shorter than 200 ms and no response trials were excluded (0.12% of trials). Figure 4b shows the mean RTs as a function of distractor condition in the single cued memory item condition in Experiment 4. There was a main effect of distractor condition, *F* (3, 105) = 44.541, *p* < 0.001, *η*_*p*_^2^ = 0.560. Further pairwise comparisons with Bonferroni adjustment revealed that the RTs of the Cued distractor condition were significantly longer than the RTs of the Uncued distractor, the New distractor condition and the No distractor condition (*ps* < 0.001). Also, the RTs of the Uncued condition was significantly longer than the RTs of the No distractor condition (*p* < 0.001). And the New distractor condition produced significantly longer RTs than the No distractor condition (*p* < 0.001). The same analysis on search accuracy showed no such effects, *F* (3, 105) = 1.045, *p* = 0.376, *η*_*p*_^2^ = 0.029 (see Table 1 for search accuracy).

Accuracy for the memory task in the single cued memory item condition in Experiment 4 is also listed in Table 1. There was no significant main effect of distractor type, *F* (3, 105) = 0.969, *p* = 0.410, *η*_*p*_^2^ = 0.027. Thus, the performance of the memory test was not affected by the different distractor conditions.

Figure 4d shows the mean RTs as a function of distractor condition in the two memory items condition in Experiment 4. There was a main effect of distractor condition, *F* (3, 105) = 27.662, *p* < 0.001, *η*_*p*_^2^ = 0.441. Further pairwise comparisons with Bonferroni adjustment revealed that the RTs of the M1 condition and the M2 condition were significantly longer than the RTs of the New distractor condition (*ps* < 0.01). Also, the RTs of the M1 condition and the M2 condition were significantly longer than the RTs of the No distractor condition (*ps* < 0.001). And the New distractor condition produced significantly longer RTs than the No distractor condition (*p* < 0.001). The same analysis on search accuracy showed no such effects, *F* (3, 105) = 2.305, *p* = 0.081, *η*_*p*_^2^ = 0.062 (see Table 1 for search accuracy).

Accuracy for the memory task in the two memory items condition is also listed in Table 1. There was no significant main effect of distractor type, *F* (3, 105) = 0.088, *p* = 0.966, *η*_*p*_^2^ = 0.003, indicating that the performance of the memory test was not affected by the different distractor conditions.

### Within-experiment Comparison

The MCI for different distractors in Experiment 4 are listed in Table 2. The M1 and M2 distractors in the two memory items condition produced an MCI comparable with that for the Cued distractor in the single cued memory item condition, both *ts* < 1.12, both *ps* > 0.27. But they both have larger MCI than Uncued distractor in the single cued memory item condition, both *ts* > 3.69, both *ps* < 0.001. These results are inconsistent with the hypothesis that M1 and M2 are alternately activated in VWM.

### Experiment 5

**Figure 5:**
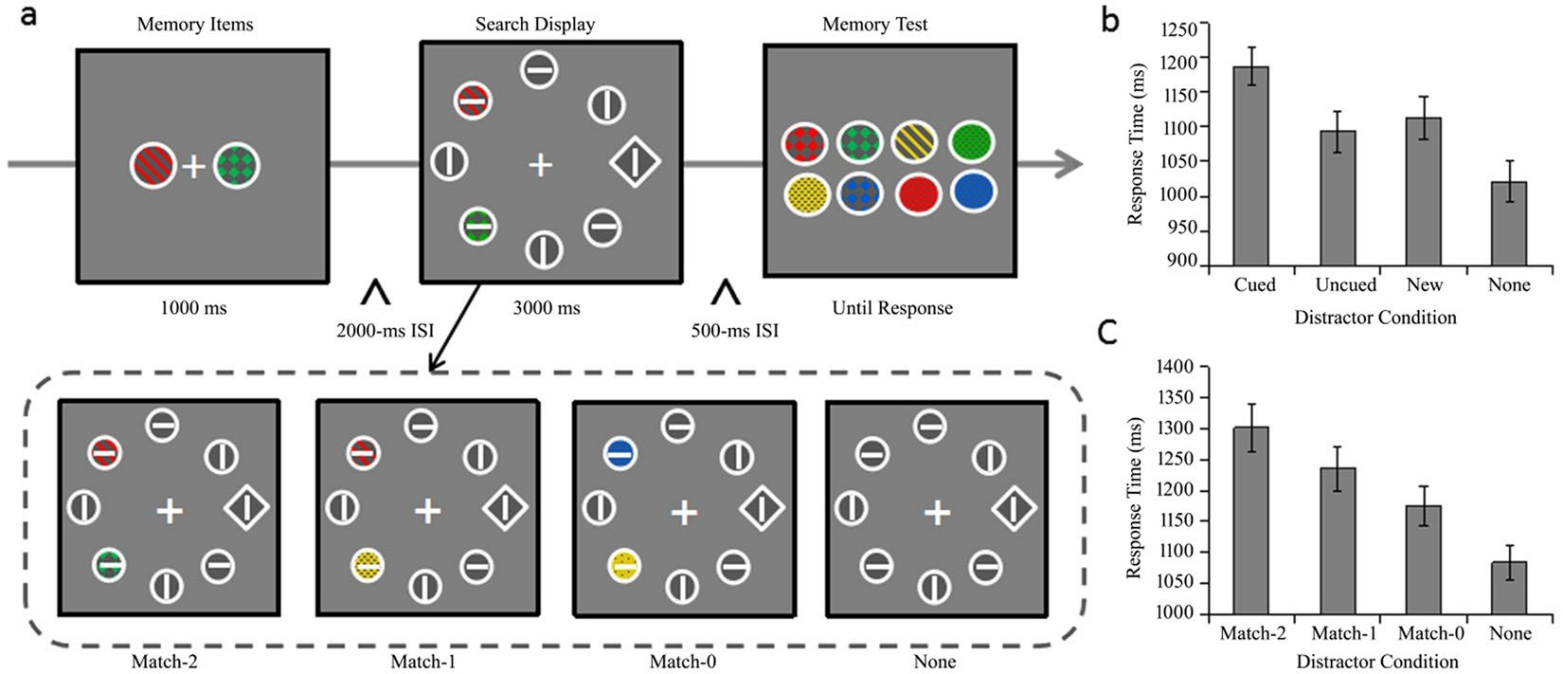
Trial sequences and results for Experiment 5. (**a**) The sequence of events with four possible distractor conditions in a trial of the two memory items condition (Match-2, Match-1, Match-0 and None distractor condition). (**b**) RTs for the search task as a function of distractor condition in the memory 1 item condition. (**c**) RTs for the search task as a function of distractor condition in the two memory items condition. Error bars indicate ±1 SE.

If M1 and M2 share a single active WM slot, the combined memory-driven capture effect for M1 and M2 should be equal to that of Cued object. In contrast, if there are multiple active slots in VWM, then both M1 and M2 can be simultaneously activated to capture attention as much as the Cued object. As a result, the combined memory-driven capture effect for M1 and M2 should be significantly larger than the Cued object. The ***Experiment 5*** aimed to examine whether the combined memory-driven capture effect for M1 and M2 is larger than the Cued object by presenting two distractors matching two memory items in visual search^27^. RTs shorter than 200 ms and no response trials were excluded (0.89% of trials). Figure 5b shows the mean RTs as a function of distractor condition in the single cued memory item condition in Experiment 5. There was a main effect of distractor condition, *F* (3, 87) = 61.547, *p* < 0.001, *η*_*p*_^2^ = 0.680. Further pairwise comparisons with Bonferroni adjustment revealed that the RTs of the Cued distractor condition were significantly longer than the RTs of the Uncued distractor, the New distractor condition and the No distractor condition (*ps* < 0.001). Also, the RTs of the Uncued and New distractor condition were significantly longer than the RTs of the No distractor condition (*ps* < 0.001). The same analysis on search accuracy showed no such effects, *F* (3, 87) = 1.307, *p* = 0.277, *η*_*p*_^2^ = 0.043 (see Table 1 for search accuracy).

Accuracy for the memory task in the single cued memory item condition in Experiment 5 is also listed in Table 1. There was no significant main effect of distractor type, *F* (3, 87) = 2.417, *p* = 0.07, *η*_*p*_^2^ = 0.077. Thus, the performance of the memory test was not affected by the different distractor conditions.

Figure 5c shows the mean RTs as a function of distractor condition in the two memory items condition in Experiment 5. There was a main effect of distractor condition, *F* (3, 87) = 44.169, *p* < 0.001, *η*_*p*_^2^ = 0.604. Further pairwise comparisons with Bonferroni adjustment revealed that the RTs of the Match-2 and Match-1 conditions were significantly longer than the RTs of the Match-0 and No distractor conditions (all *ps* < 0.001), and more importantly, the RTs of the Match-2 condition were significantly longer than the Match-1 condition (*p* = 0.002). And the Match-0 distractor condition also produced significantly longer RTs than the No distractor condition (*p* < 0.001). The same analysis on search accuracy showed no such effects, *F* (3, 87) = 2.351, *p* = 0.078, *η*_*p*_^2^ = 0.075 (see Table 1 for search accuracy).

Accuracy for the memory task in the two memory items condition is also listed in Table 1. There was a main effect of distractor type, *F* (3, 87) = 4.08, *p* = 0.009, *η*_*p*_^2^ = 0.123, further pairwise comparisons revealed that the memory performance of the Match-0 condition were significantly lower than the None condition (*p* = 0.039).

### Within-experiment Comparison

The MCI for different distractors in Experiment 5 are listed in Table 2. The Match-2 produced a larger MCI than the Match-1 and the Cued distractor condition, both *ts* > 2.09, both *ps* < 0.05. Also, the Match-1 and the Cued distractor condition both have a larger MCI than Uncued distractor in the single cued memory item condition, both *ts* > 6.95, both *ps* < 0.001. The MCI for Match-1 distractor in the two memory items condition was not different from that for the Cued distractors in the single memory item condition, *t* = 1.306, *p* = 0.202. These results indicated that M1 and M2 are simultaneously activated in VWM.

### Between Experiments Comparison

We also compared the MCIs for the different distractor conditions across Experiments 1–5 (see Table 2). If the M1 and M2 were alternating as the sole active representation in VWM, then they should produce a smaller MCI than the single cued memory item condition of Experiments 2, 4 and 5. However, most M1 and M2 distractors (six out of eight) in Experiments 1, 3 and 4 produced a numerically larger MCI than the Cued distractor condition in Experiment 2, although no difference was significant, all *ts* < 0.534, all *ps* > 0.596. Similarly, the M1 and M2 distractors in Experiments 1–3 produced a MCI comparable to that for the Cued distractor condition in Experiment 4, all *ts* < 1.65, all *ps* > 0.102. And likewise, the M1 and M2 distractors in Experiments 1–4 produced comparable MCI as that for the Cued distractor condition in Experiment 5, all *ts* < 1.50, all *ps* > 0.14. Thus, the present results are consistent with the hypothesis that M1 and M2 are simultaneously activated in VWM.

## Discussion

The five experiments in the present study consistently showed that when memorized items include a conjunction of two features, a distractor matching either of the two memorized representations can capture attention and interfere with concurrent visual search. Since the two memorized items were randomly chosen from multiple possible items (eight possibilities in Experiment 1, or twelve possibilities in Experiments 2–5) and varied from trial to trial, it is unlikely that the memorized items were stored in long-term memory^15, 18^. In addition, the two VWM representations consistently produced a memory-driven capture effect that was comparable to a single cued representation in VWM. This is the first observation that two VWM representations, which are irrelevant to the concurrent visual search, can be sufficiently active to capture attention, directly affecting the visual search. It is in sharp contrast to all the previous findings that memory-driven capture is absent when participants are required to memorize two objects^14, 28, 29^. Thus, it undermines the claim that only one VWM representation can be active in guiding attention^13, 14^.

It might be argued that two VWM representations alternately activate to influence concurrent visual search. If two VWM representations share a single active WM slot, only one (either M1 or M2) can be activated to guide attention on any given trials. As a result, the combined memory-driven capture effect for M1 and M2 should be equal to that of Cued object. Furthermore, at least one of them should produce a memory-driven capture smaller than that for the Cued distractor. However, these hypotheses are inconsistent with the present findings. First, when two memory-matching distractors appear in visual search (Match-2 conditions in Exp 5), they produced significantly larger Memory-driven capture than a single cued memory item in Experiment 5. Second, for the same group of participants in Experiment 2 and Experiment 4, the memory-driven capture effect by a single cued item was not different from the capture effect driven by either of two memory items. In addition, the memory-driven capture effect by a single cued item was also comparable to the capture effect by either of two VWM representations in Experiments 1 and 3. In contrast, if there are multiple active slots in VWM, then M1 and M2 can be simultaneously activated in VWM as a single Cued object does. As a result, the combined memory-driven capture effect for M1 and M2 should be larger than that of Cued object. Additionally, both M1 and M2 are supposed to capture attention as much as the Cued object. Therefore, present results suggested that either of two VWM representations simultaneously guide attention, interfering with concurrent visual search as much as a single memorized item.

The present results suggest that VWM can have two representations automatically activated to guide attention at same time^20^. Whether multiple VWM representations can guide attention depends on how active those VWM representations are. If multiple VWM representations are strong enough, they can have simultaneous control of attention, influencing perceptual selection directly. Previous studies have shown that neural representations for a feature conjunction are enhanced relative to those for a single feature^25^. These findings might explain why two VWM representations for the feature conjunction are sufficiently active to guide attention, whereas two VWM representations for the individual feature usually cannot influence attention^14^. Similarly, when two VWM representations are assigned as target templates, they are highly prioritized and influence perceptual processes^20, 23, 24^. In addition, whether multiple VWM representations can guide attentions might also depends on the demand characteristic of search task. For example, a most recent study also showed that two VWM representations of color can simultaneously capture attention in the gap-location task but not in the shape-singleton task^27^. However, the present results showed that two VWM representations for the feature conjunction are sufficiently active to guide attention even in a shape-singleton task, indicating the special role of VWM representations for a feature conjunction.

At first glance, the present finding is just replicating the previous findings that two target templates can simultaneously control attention^20, 23, 24^. However, it is actually different from those previous findings for two reasons. First, the attentional priority was involuntarily assigned to two VWM representations in the present study since they were totally irrelevant to the visual search. In contrast, when the memorized items are target templates, they receive voluntary attention. Second, although targets changed from trial to trial^20, 24^ or even varied within a single trial^23^, there were only two possible target colors, thus resulting in two constant target templates in those earlier studies. A recent study showed that two constant target templates are stored in long-term memory rather than VWM^17^. Thus, the present study is the first to show that two irrelevant VWM representations can be simultaneously activated to capture attention in a singleton search task.

Previous research also suggested that VWM representations are more likely to interfere with concurrent visual search when the retention interval is relatively short, probably due to a stronger representation at short intervals^26^. Since the SOA was relatively short in Experiments 1–2 of the present study, it might partially contribute to the memory-driven capture effect by two VWM representations. However, since two VWM representations also produced a large memory-driven capture effect with an extended SOA in Experiments 3 and 4, the SOA per se cannot account for why two VWM representations simultaneously control attention. Moreover, Experiments 1–3 showed longer RTs than those reported in previous studies^14, 27^. The generally longer RTs in Experiments 1–3 might be due to the difference in the search task. In previous study, the search target is a horizontal or vertical bar within the diamond^14, 27, 30^, while Experiments 1–3 here set either “N” or “M” as the search target. In Experiment 4, we asked participants to search for a horizontal or vertical bar as Moorselaar *et al*.s’ study^14^, results showed that RTs were faster than those in Experiments 1–3 and comparable with previous studies^14, 30^. More importantly, each of two VWM representations still produced a large memory-driven capture effect. These results further suggest that two VWM representations can simultaneously control attention.

Admittedly, the memory-driven capture here might be partly due to participants’ strategy of attending to working memory content for better memory performance. Kiyonaga, Egner, and Soto’s study^31^ have shown that “attentional capture by WM contents is partly, but not fully, malleable by top-down control”. However, the present study argued that multiple working memory representations, instead of a single one, can be simultaneously activated to capture attention. Whether the simultaneous capture by two VWM representations is *partly* boosted by participants’ strategy of attending to working memory contents cannot invalidate our demonstration of the simultaneous capture of two VWM representations. More specifically, if there is only one active VWM slot, no matter how hard participants tried, only one VWM representation can capture attention, or two VWM representations alternatively capture attention. However, both present results and Hollingworth & Beck study^27^ convincingly refute these possibilities. Thus, as long as we show that two working memory representations can be simultaneously activated to capture attention, the single active slot theory should be rejected.

In summary, the interface between attention and visual working memory is not as extremely capacity-limited as we used to believe. Two VWM representations can simultaneously pull attention away from concurrent visual search even though they are irrelevant to the search task.

## Method

### Participants

Twenty five naive students participated in Experiment 1 (13 females; aged 19–26 years). However, one participant was excluded because his accuracy on the memory task was less than 60%. Forty naïve students participated in Experiment 2 (20 females; aged 18–29 years) and two participants were excluded because their mean accuracy on the memory task was less than 60%. Twenty four naïve students participated in Experiment 3 (14 females; aged 18–26 years). Thirty eight naïve students participated in Experiment 4 (20 females; aged 18–29 years). Thirty naïve students participated in Experiment 5 (19 females; aged 18–26 years). All participants had normal or corrected-to-normal vision and color perception and were paid for money after the experiments. Written informed consent was provided by each participant prior to the experiments. All experimental methods were conducted in accordance with the approved guidelines. The study was approved by the Research Ethics Committee of the Institute of Psychology, Chinese Academy of Sciences.

### Materials and Procedure

All stimuli were presented on a 17-inch CRT monitor at a viewing distance of approximately 60 cm. The monitor was set to a 1024 × 768 resolution with an 85 Hz refresh rate. The font was Arial. All stimuli were presented on a black background.

#### Experiment 1

The sequence of trials in Experiment 1 is illustrated in Fig. 1a. In each trial, an instruction to “remember two items” appeared at the center of the screen for 500 ms. This instruction was followed by two disks with different conjunctions of color and texture which also lasted for 500 ms. The colors of the two disks were randomly chosen from four colors: red (RGB: 250, 20, 0), green (RGB: 0, 170, 0), yellow (RGB: 220, 200, 20), or blue (RGB: 0, 90, 200). One disk was a solid disk; the other was a disk with stripes. Each disk had a radius of 0.6° and was positioned at 2.5° either left or right of the central fixation cross. A 300 ms blank screen was presented after the two disks disappeared; this was followed by a search display. The search display consisted of a gray diamond (1.2° in size) and seven disk distractors (each with a radius of 0.6°). They were placed on the rim of an imaginary circle (with a radius of 8°), which was centered on the fixation. The diamond contained a black target letter which could be either an “N” or an “M” (0.38° in size). Each disk distractor contained a symbol resembling an hourglass. Six of the seven disks were in solid gray and the other one was in one of four possible combinations of color and texture: (1) In the M1 distractor condition, the disk’s color and texture was the same as the memorized items on the left; (2) In the M2 distractor condition, the disk’s color and texture was the same as the memorized items on the right; (3) In the New distractor condition, the disk was a new solidly colored disk, which was not a memorized item; (4) In the No-distractor condition, all seven disks were gray (RGB: 85, 85, 85). Participants were instructed to indicate whether the diamond contained “N” or “M” as fast as possible. The diamond and color distractor never appeared in adjacent positions in the search display. The search display was present until response and was then followed by another 500 ms blank screen.

After the blank screen, a probe disk (with a radius of 0.6°) appeared at the center of the display. Participants were required to report whether the probe disk matched with either of the memorized disks. On half of the trials, both the color and texture of the probe matched one of the memorized disks. On another quarter of the trials, the probe disk matched with the memorized disks in color but they differed in texture. For the remaining quarter of the trials, the probe disk matched with the memorized disks in texture but they differed in color. All participants completed 12 practice trials and four blocks of 40 trials in Experiment 1. The four distractor conditions were equally distributed within each block.

#### Experiment 2

The events are illustrated in Fig. 2a and c. The materials and procedure were identical to Experiment 1 with three exceptions. First, since the solid disk in Exp 1 might be perceived as a color disk without texture, the solid texture was excluded in Exp 2. Thus, the two memory items in Experiment 2 were drawn from 12 possible combinations of four colors and three types of texture (checkboard, striped and reticulation). Second, in memory task the present experiment asked participants to report whether the memorized disks were present among 8 probe disks. Some probe disks might either share the same color but differ in texture or share the same texture but differ in color as the memorized item, therefore participants cannot use a single feature for memory task. Third, two memory conditions were tested for each participant. They were either required to memorize one cued item in the single cued memory item condition, or to memorize two items in the two memory item condition. In the single cued memory item condition, a gray arrow cue (RGB: 85, 85, 85; 0.8° in width, 1.6° in length; pointing either to the right or left) was above the two memory items, and participants were explicitly instructed to memorize the Cued item and then report whether the Cued item was present in the memory test. The Cued disk was only presented on a half of the trials. The Uncued disk never appeared as a probe disk. However, in the two memory item condition, observers were instructed to memorize both items and then report whether one of the two memorized disks was present among the probes. M1 and M2 were present in the probes with equal probability for 50% of the trials. They never occurred in the probe display simultaneously. The order of the two memory conditions was counter-balanced across participants.

Finally, the four distractor conditions in the visual search were slightly different for the two memory conditions. In the single cued memory item condition, the four distractor conditions were: Cued, Uncued, New and None (illustrated in Fig. 2a). In contrast, in the two memory items condition, the four distractor conditions were: M1, M2, New and None condition (illustrated in Fig. 2c). All participants completed 12 practice trials and two blocks of 96 trials for each of the two memory conditions, resulting in a total of 24 practice trials and 384 experimental trials.

#### Experiment 3

The events are illustrated in Fig. 3a. The procedure was identical to those for the two memory items condition in Experiment 2 with two exceptions. First, the memory display was presented for 1000 ms. Second, the retention interval between the memory display and the search display was 2,000 ms. All participants completed 12 practice trials and two blocks of 96 trials (altogether 192 testing trials).

#### Experiment 4

The events are illustrated in Fig. 4a and c. In order to closely mimic the study of Moorselaar *et al*.^14^, the material and procedure in Experiment 4 were identical to Experiment 2 with four changes. First, the search display consisted of a gray diamond (2.6° in size) and seven disks (each with a radius of 1.5°). They were placed on the rim of an imaginary circle (with a radius of 8°), which was centered on the white fixation (0.3° * 0.3°). The diamond contained either a horizontal or vertical bar, and participants were instructed to report whether the diamond contained a horizontal or a vertical line as fast as possible. Second, as Moorselaar *et al*. study^14^, memory items and probe stimulus (each with a radius of 1.5°) had white outline. Third, the search display remained present for a maximum of 3 sec or until a response was made. Since most excluded RTs in previous experiments distributed above 3 s, we set up a 3 s deadline to urge participants respond as soon as possible. Fourth, as in Experiment 3, the memory display was presented for 1000 ms, followed by 2000 ms retention interval.

Participants receive 80 trials of the search task as pre-practice. Then all participants completed 12 practice trials and two blocks of 96 trials for each memory condition in Experiment 4, resulting in a total of 24 practice trials and 384 experimental trials.

#### Experiment 5

The single cued memory item condition was same as that in Exp 4. There were four distractor conditions in visual search for two memory items condition: Match-2, Match-1, Match-0 and None conditions (illustrated in Fig. 5a). In the Match-2 condition, two memory-matching items appear as distractors in visual search. In the Match-1 condition, one memory-matching item appears as distractors in visual search and another distractor is a new item. In the Match-0 (New) condition, two distractors in visual search were new items. In the None condition, all disks were gray.

Participants completed 12 practice trials and two blocks of 96 trials for each of the two memory conditions, resulting in a total of 24 practice trials and 384 experimental trials.
